# Evidence for the Effectiveness and Acceptability of e-SBI or e-SBIRT in the Management of Alcohol and Illicit Substance Use in Pregnant and Post-partum Women

**DOI:** 10.3389/fpsyt.2021.634805

**Published:** 2021-05-05

**Authors:** Trecia A. Wouldes, Andi Crawford, Suzanne Stevens, Karolina Stasiak

**Affiliations:** ^1^Department of Psychological Medicine, Faculty of Medical and Health Science, University of Auckland, Auckland, New Zealand; ^2^Te Ara Manapou, Parenting and Pregnancy Service, Hawke's Bay District Health Board, Hastings, New Zealand

**Keywords:** pregnancy and post-partum, alcohol and illicit drug use, screening and brief intervention, mobile health or mHealth, barriers to treatment

## Abstract

Alcohol and illicit psychoactive drug use during pregnancy have increased worldwide, putting women and their children's health and development at risk. Multiple drug use, comorbid psychiatric disorders, sexual and physical abuse are common in women who use alcohol and drugs during pregnancy. The effects on the mother include poor reproductive and life-long health, legal, family, and social problems. Additionally, the exposed child is at increased risk of long-term physical health, mental health, and developmental problems. The stigma associated with substance use during pregnancy and some clinicians' reticence to inquire about substance use means many women are not receiving adequate prenatal, substance abuse, and mental health care. Evidence for mHealth apps to provide health care for pregnant and post-partum women reveal the usability and effectiveness of these apps to reduce gestational weight gain, improve nutrition, promote smoking cessation and manage gestational diabetes mellitus, and treat depression and anxiety. Emerging evidence suggests mHealth technology using a public health approach of electronic screening, brief intervention, or referral to treatment (e-SBIRT) for substance use or abuse can overcome the typical barriers preventing women from receiving treatment for alcohol and drug use during pregnancy. This brief intervention delivered through a mobile device may be equally effective as SBIRT delivered by a health care professional in preventing maternal drug use, minimizing the effects to the exposed child, and providing a pathway to therapeutic options for a substance use disorder. However, larger studies in more diverse settings with women who have co-morbid mental illness and a constellation of social risk factors that are frequently associated with substance use disorders are needed.

## Introduction

The number of mobile phone users in 2020 is 5.22 billion, which represents 66.8% of the world population of 7.81 billion. Of these, 3.5 billion are smartphone users, up from 2.5 billion in 2016, and is estimated to be 3.8 billion in 2021 (1). With this increase has come an increase in the use of technology to adopt healthy lifestyles or support medical health care through mobile health applications (mHealth apps). At present there is no single accepted definition of mHealth. The World Health Organization (WHO) defines mHealth as “the use of mobile devices (mobile phones, personal digital assistants, or patient monitoring devices) for medical and public health practice” (2). The National Institutes of Health defines mHealth as the use of mobile and wireless devices to improve health outcomes, health care services and health research and includes wireless devices such as mobile phones, tablet computers and PDAs as well as patient monitors and electronic health records. A growing number of mHealth apps are being developed that target personal lifestyle and health care support (3). Among these are mHealth apps that focus on common health issues during pregnancy such as gestational diabetes, nutrition, depression and anxiety (4, 5).

The use of alcohol and illicit recreational drugs by women during pregnancy has increased worldwide and represents a significant risk to the health and mental health of these mothers and the health and development of their children (6–11). Pregnancy may be considered a “window of opportunity,” where research has shown that women are more open to changing behaviors, such as smoking and consuming alcohol, to protect their baby (12, 13). Yet, those who need care for substance misuse during pregnancy often do not receive it because they don't think they need it (14, 15), or because of the stigma of maternal drug use, involvement with the criminal justice system, and the threat of loss of child custody (16–18). Additionally, a variety of barriers in the health care system may prevent women from getting information about the effects of alcohol and substance use on their pregnancy or obtaining specialist care (16, 19–28). Some of these barriers reported by clinicians include competing priorities and time constraints, lack of knowledge or conflicting evidence about the effects on the baby, and lack of knowledge about screeners (19, 29–31). This report will provide an overview of the prevalence and adverse consequences of alcohol and common drugs of abuse, and the barriers that interfere with women obtaining prenatal care that includes support to reduce substance use. Emerging evidence suggests that technology may provide the answer to overcoming these barriers (32–42). The focus of this report will be the usability and effectiveness of mobile health technology in providing prenatal (and perinatal) care for substance use disorders. The structure of the report is as follows: first, we will address important background information regarding the prevalence of alcohol and drug use by women of child-bearing age, the consequences of alcohol and drug use during pregnancy, and the barriers to screening during pregnancy from the perspective of women who have used drugs during pregnancy and the clinicians involved in their care; second, we will review the current evidence for the effectiveness of SBIRT delivered through mHealth technologies. Finally, the section Discussion will explore the implications of the ability of mHealth technology to potentially provide a more equitable public health approach to treating alcohol and drug use in this high-risk population through the lens of current evidence.

## Background

### Prevalence of Alcohol and Common Illicit Recreational Drugs in Women of Child-Bearing Age

#### Alcohol

An estimated 2.3 billion people consumed alcohol worldwide and projected estimates point to increased global consumption in the next 10 years, particularly in the South-east Asia and Western Pacific regions and the Americas. Overall, 5.1% of the global burden of disease and injury is attributable to alcohol, with 13.5% of deaths in young people associated with alcohol consumption (43). Of particular concern is the extent of alcohol use by women of childbearing age. The global estimate of the prevalence of alcohol use during pregnancy is 9.8%, with the highest prevalence estimated to be 25.2% in the WHO European region (44). Global estimates of binge drinking during pregnancy in the general population range between 0.2 and 13.9% (45). In the absence of moderate daily consumption, binge drinking has been linked to an increased risk for mental health problems, especially hyperactivity and inattention in prenatally exposed children (46). The regions with the highest proportion of women who binge drink during pregnancy include both higher income and lower and lower middle-income countries (African, European, Americas, and Western Pacific). The countries with the highest estimated prevalence of binge drinking of those women who consumed alcohol were Paraguay (13.9%), Moldova (10.6%), Ireland (10.5%), and Lithuania (10.5%).

#### Cannabis

Cannabis is still considered illegal in most countries and worldwide, is the most commonly used illicit substance in general and pregnant populations (47, 48). In 2018, 192 million people used cannabis (47). Regional trends in people aged 15–64 indicate high rates in West and Central Africa (12.4%), North America (12.1%), Columbia (15.2%), and Oceania (10.8%). Approximately 13.1 million people are cannabis-dependent globally (49). Males have a higher rate of cannabis dependence (0.20–0.27%) than females (0.12–0.16%). However, women exhibit an accelerated progression to a cannabis use disorder. A scoping review of cannabis use in high-income countries including the United States (US), Australia, the United Kingdom, Canada, France, and the Netherlands found the prevalence of prenatal cannabis use ranged from 0.24 to 22.6% (50). Prenatal cannabis users across studies appeared to be younger than 25 years of age, of low parity, and single compared to non-users. Also, cannabis users were more likely to have a lower income and be less well-educated. Consistent with the rise in the prevalence of cannabis use in the general population, and regardless of legalization, the prevalence of prenatal cannabis use appears to be rising during pregnancy (51). For instance, in one US study the prevalence of use during pregnancy increased from 3.4 to 7.0% from 2002–2003 to 2016–2017 (52). In a Canadian study, cannabis use increased from 2.2 to 3.3% between 2008 and 2016 (53). In one further study, prenatal use increased from 4.2% in 2009 to 7.1% in 2016 with a higher annual rate in women whose use was confirmed by a urine toxicology test compared to a self-report group (54).

#### Prescription Opioids, Heroin, and Opioid Substitution Treatment

In 2018, an estimated 57.8 million people used opioids globally, including opiates such as heroin and opium, pharmaceutical and other synthetic opioids (47). Of the estimated 167,000 deaths attributed to drug use disorders in 2017, 66% were due to opioid use and accounted for nearly 80% of the 42 million years of “healthy” life lost due to disability and pre-mature death (47). The recent surge in the maternal use of opioids is largely attributable to the sale of opioid pain relievers in the US, with 9.5–41.6% of women filling a prescription in 2007 across six states. Prenatal opioid use and abuse of prescription opioids, and more recently heroin resulted in a documented 333% increase in fetal exposure between 1999 and 2014 in the US. Between 2000 and 2010, US national estimates suggest a 35% increase in opioid prescriptions dispensed. Increases were also seen in Norway where 6% of pregnant women filled a prescription between 2004 and 2007 (55, 56). While there is a lack of prevalence data for maternal prescriptions of opioid pain relievers elsewhere, there are reports of increased use of prescription opioids in Australia (57), New Zealand (58), Canada (59), Germany (60), Israel (61), and the United Kingdom (62). The best evidence for the increased abuse of opioids during pregnancy comes from reports of a five-fold increase in neonatal abstinence syndrome (NAS) (63, 64), increased referrals to neonatal intensive care units (NICU), and extended hospitalizations of exposed infants postnatally (63). Given the increase in opioid use and dependence, opioid substitution treatment may be prescribed, predominantly methadone and buprenorphine which are synthetic opioids. Despite the importance of these drugs in minimizing the effects of illicit opioids on the mother and her baby, both have been associated with NAS and methadone has been implicated in poorer clinical outcomes for the prenatally exposed child compared to the unexposed child (65).

#### Stimulants—Cocaine and Methamphetamine

In 2018, it is estimated that 27 million people worldwide used amphetamine type stimulants, predominantly crystalline methamphetamine, and 19 million reported past year use of cocaine (0.5 and 0.4% of the global population, respectively) (47).The main cocaine markets continue to be North America, West and Central Europe. North America has the highest prevalence of methamphetamine use (2.3%), followed by Australia and New Zealand (1.3%). Although, the use of cocaine and methamphetamine by women during pregnancy has become a significant public health concern over the past three decades the prevalence of stimulant use worldwide is hard to estimate (8). Available evidence indicates that stimulant use in pregnancy differs by region in the US and globally which is consistent with the pattern of use in the general population (66). Cocaine use during pregnancy, in one US report, estimated the past month's use was 0.2% of women in the US. An early estimate in 2006 from a US study found 5.2% of an unselected sample of 1,632 women reported using methamphetamine during pregnancy (67). A further indication of the rise in the abuse of methamphetamine and cocaine during pregnancy in the same period come from reports on increased admissions to treatment for substance abuse associated with stimulants. For instance, admissions tripled in federally funded programs for methamphetamine dependence from 8% in 1994 to 24% by 2006 (68). Recent reports in the media (69, 70) and evidence from drug reporting and protection agencies suggests a resurgence of cocaine and methamphetamine use in the general population and women of childbearing age in the US and worldwide (8, 71).

#### Multiple Drug Use

Women who continue to use any drugs during pregnancy are also more likely to use a combination of other psychoactive substances. In 8 of 10 studies of prenatal cannabis users compared to non-users, alcohol, tobacco, and illicit drug use was higher among users than non-users (50). Studies investigating methamphetamine and cocaine use during pregnancy found women who used these substances were also more likely to use significant amounts of alcohol, cannabis, and continue to smoke tobacco throughout their pregnancy than a matched control group (67, 72–75). Women who report using opioids during pregnancy and women who are receiving methadone or buprenorphine to treat their opioid dependence during pregnancy also report more multiple drug use including more frequent use of cannabis, tobacco, stimulants and benzodiazepines throughout their pregnancy and more use and misuse of prescribed medications (76–78).

### Consequences of Alcohol and Illicit Drug Use During Pregnancy

#### Alcohol

All recreational drugs consumed during pregnancy cross the placenta, and depending on the drug, the dose, timing, and frequency of use may damage the brain and other organs of the developing fetus (6–10). At present there is no known safe level or timing of drinking for pregnant or lactating women, however, binge drinking (≥4 drinks within 2 h or a blood alcohol level of 0.08 g/dl or above) or chronic heavy drinking (≥3 drinks per day) are reported to have the most harmful consequences (6, 45). Alcohol use during pregnancy is associated with negative birth outcomes that include miscarriage, still birth, low birth weight, pre-term birth, and intrauterine growth retardation (6). Alcohol exposure can also lead to physical, cognitive, and behavioral impairments collectively known as fetal alcohol spectrum disorder (FASD). At the extreme end of the spectrum heavy exposure could result in fetal alcohol syndrome (FAS) which is associated with characteristic facial features, poor growth, irreversible neurological problems, developmental delay, seizures and brain deformations (6, 79). Of particular concern is the life-long adversity that may occur due to the effects of prenatal alcohol exposure. In a recent global study, the prevalence of FASD was 10–40 times higher in specialized clinical services, correctional institutions, children in out-of-home care and special education programmes, and in Aboriginal populations than the 7.7 per 1,000 global prevalence (80).

#### Cannabis

Recently, cannabis use has become more prevalent among pregnant and breastfeeding women due to the increasing social acceptability of its use, the perception that it is safe, and reports that it reduces nausea and depression with no adverse consequences for the mother and child (81, 82). Early research found cannabis use during pregnancy was associated with still birth, fetal growth restriction, and neurodevelopmental consequences (83, 84). However, much of this and current research suffers from a number of methodological limitations such as not controlling for other drug use or only measuring cannabis use at one time point (50). A recent meta-analysis of child health outcomes at birth found cannabis-exposed infants had lower birth weights than non-exposed infants and were more likely to be referred to a NICU (7). In contrast, data from the US Pregnancy Risk Assessment Monitoring System (*N* = 4,969) that included women from states where recreational use of cannabis was legal and others where it wasn't, found no association between cannabis use and lower birth weight after controlling for relevant covariates such as cigarette or tobacco use (9). Unspecified in this study was the quantity or frequency of cannabis use, the form or method of use, and the quantity of delta-9-tetrahydorcannibol (THC) which is the psychoactive ingredient in cannabis. Current research has shown THC has increased as have the variety of forms (e.g., candies, drinks, suppositories, snacks, lollipops, and capsules) and the methods of use (e.g., vaporizing, skin absorption, oral consumption), and the frequency of use. All of which are likely to increase the exposure to the developing fetus (85).

#### Opioids

Daily opioid substitution treatment during pregnancy with methadone or buprenorphine has been found to be beneficial for the mother in reducing illicit use and illegal activities, improving antenatal care, and reducing maternal craving and withdrawal (86–89). Opioid substitution treatment has also been shown to be more beneficial for the developing fetus compared to heroin and other illicit opioids, but all opioids along with prescription opioids have been associated with poorer outcomes for the exposed infant (11, 65, 77, 78, 90, 91). Maternal opioid use is associated with an increased risk of NAS or neonatal opioid withdrawal syndrome (NOWS). This occurs when opioid exposure *in utero* triggers withdrawal at birth when the infant is separated from its supply of opioids. NOWs occurs in anywhere from 45 to 95% of infants exposed to opioids, including opioid substitution drugs such as methadone and buprenorphine (65, 77, 78). Signs of opioid withdrawal include evidence of CNS irritability, gastrointestinal dysfunction, yawning, sneezing, and fever. Behaviorally, babies experiencing abstinence frantically suck their fingers, display incessant and inconsolable high-pitched crying and are restless and irritable (92). In cases where infants are exhibiting significant and ongoing signs of abstinence, treatment with morphine to reduce symptoms may be required along with referral to special care baby units and extended hospital stays (63). Opioid exposure prenatally is also associated with postnatal growth deficiency, microcephaly, neurobehavioral problems, and sudden infant death syndrome (77, 78, 93). The evidence for the ongoing development of these children is limited because the extant literature has a number of methodological limitations including, small numbers, retrospective designs, and a lack of control of maternal psychosocial factors that could explain some of the poor outcomes [see (91, 94, 95) for comprehensive reviews]. However, results from one, recent well-designed longitudinal study has shown that infants born to mothers receiving daily doses of methadone to treat their opioid dependence had poorer outcomes on standardized measures of cognitive ability and more educational delay at 9.5 years of age than unexposed children (96).

#### Stimulants, Cocaine, and Methamphetamine

Early reports of prenatal cocaine and methamphetamine exposure suggested infants were at risk of fetal malformations and anomalies. However, many of these reports did not control for multiple drug use or the associated psychosocial or obstetric risks that could also explain these results (8, 10). More recently, well-designed longitudinal studies controlling for other drug use and the postnatal environment of exposed children have found less deleterious outcomes. Notwithstanding, after controlling for other drug use and a wide range of psychosocial factors, prenatal exposure to both methamphetamine and cocaine have been associated with being born preterm, decreased birth weight, length and head circumference [see (97–100) for reviews]. Catch-up growth in cocaine exposed children has been reported by 6 years of age (98). Catch up in weight and head circumference has been found for methamphetamine-exposed children but not height (101). In a cross-national study, a stronger negative effect of methamphetamine on infant and child length/height was reported for US children compared to New Zealand (NZ) children (101). Unlike opioid exposure, no clear withdrawal syndrome has been observed in stimulant exposed children, but both cocaine and methamphetamine exposure are associated with disturbed neurobehavior at birth and 1 month postnatally (98, 102). Poorer state regulation, quality of movement, lower arousal, and increased CNS stress were observed in exposed children compared to non-exposed comparison groups. Notable is the association between poor neurobehavior in this population and poorer medical, behavioral, cognitive and motor problems at 12, 24, 36, and 48 months in children in the US and in NZ (103, 104). Early cognitive outcomes of both cocaine and methamphetamine exposed children have generally been associated with lower scores on global IQ tests when compared to those of non-exposed, but differences were often explained by other adverse environmental and drug exposures. More recently, however, tests of specific neuropsychological capabilities have shown a range of adverse effects on executive function, inhibitory control, working memory and attention (100). The effects of prenatal cocaine exposure long term are associated with more delinquent behavior, more externalizing behavior, poorer mood, a greater risk for substance use related problems and early onset and riskier sexual behavior by age 17 (105). While there is evidence for the long-term outcomes of cocaine exposure on child development, little is known about the effects of methamphetamine past middle childhood. The best evidence for the effects of cocaine and methamphetamine come from recent well-designed, longitudinal studies that demonstrate the complex interplay between prenatal exposure and the postnatal environment on child outcomes [see (10, 98) for review].

#### Maternal Consequences for Alcohol and Illicit Drug Use During Pregnancy

Mothers who use illicit drugs are at significant risk for a variety of obstetric problems such as miscarriage, pre-term labor, preeclampsia and they often report multiple elective terminations. Maternal opioid use is associated with a number of negative pregnancy outcomes that include oligohydramnios, preeclampsia, placental insufficiency and abruption, pre-mature rupture of membranes, pre-term labor, post-partum hemorrhage, and stillbirth (11, 90). Cocaine has been shown to cause maternal hypertension, vasoconstriction, and decreased uterine blood flow, leading to impaired nutrient delivery and oxygen exchange for the fetus (8). However, the longer half-life and the broader target sites in the CNS of methamphetamine mean there may be more severe outcomes for the mother and the exposed fetus. In a study of 18,050 women who used methamphetamine and opioids during pregnancy compared to the general population there were high rates of obstetric risks, but the highest rates were associated with methamphetamine use during pregnancy; these included preeclampsia, placental abruption, pre-term delivery (<37 weeks), cesarean delivery and severe maternal morbidity and mortality (106). In contrast, there is little evidence that these obstetric problems are associated with women who use cannabis, however, five studies have found women who smoked cannabis during pregnancy had increased odds of being anemic during pregnancy (7).

Finally, misuse of maternal alcohol or illicit drugs worldwide is associated with inadequate prenatal care, poor nutrition, poorer educational attainment, increased rates of unemployment, benefit dependence, mental illness, a history of sexual and physical abuse, and ongoing domestic violence (7, 8, 17, 72, 76, 107). Children who live in environments, particularly in the first 1,000 days, where there is maternal mental illness or poverty, without any substance use, are at risk of poorer health, mental health, and developmental outcomes (108). Children exposed prenatally to alcohol and/or drugs are also likely to be exposed to a living environment where there are similar as well as additional risks common to maternal drug use, further impacting their health and development.

### Barriers to Screening and Treating Alcohol and Illicit Substance Use During Pregnancy

#### Reticence for Clinicians to Screen for Alcohol and Illicit Substance Use in Pregnant Women

Clinicians involved in maternity care are well aware of the detrimental effects alcohol and illicit drug misuse can have on the mother and her developing child, however, they also report a number of barriers that prevent them from screening and providing referrals for substance use in this high-risk population. Clinicians report they are less likely to ask about alcohol and drug use at the first prenatal visit as they feel their priority at that time is building rapport (19, 20, 26). They are also less likely to ask about drug use when women are from particular ethnic or socioeconomic groups they perceive to be less likely to use drugs, or if a family member is present during a prenatal visit (19, 23, 109). There is also a perception among clinicians that most women do abstain from alcohol and illicit drug use, and they worry inquiring about use may create guilt or anxiety and, in turn, interfere with the clinical relationship (21, 26, 29). Further barriers include competing priorities, and time constraints, lack of knowledge about screeners, and no clear referral pathway (19). Lack of knowledge or conflicting evidence for the effects of prenatal exposure on the developing fetus and mother mean clinicians don't ask about common illicit drugs including, cannabis, methamphetamine or opioids, as they report they won't know what to tell the mothers about the effects (19, 29–31). One study found that clinicians were more comfortable asking about smoking than illicit drug use or common related risks to the mother, such as intimate partner violence and mental illness (19, 110). In another study, obstetricians focused more on the legal aspects of cannabis use during pregnancy (24) as they weren't aware of the health risks and did not perceive cannabis to be as unsafe as other illicit drugs. In addition to more knowledge around illicit substances and their effects, clinicians reported the need for more training around screening skills and protocols and suggested electronic screening mechanisms would be useful (19, 20, 111, 112).

#### Multiple Risks Associated With Women Reporting Alcohol or Drug Use or Seeking Treatment

Women who are pregnant may under-report their alcohol and drug use due to the stigma of drug use, lower socioeconomic status, involvement with the criminal justice system, and the threat of loss of child custody (16–18). Research suggests that maternal substance use may be under-reported even when obtained *via* interviews with highly trained staff who were not affiliated with participants' health care or social services and with whom a rapport had been established (16). Women who are asked about alcohol use after awareness of pregnancy tend to report less alcohol use than a direct biomarker of alcohol metabolism collected from newborn dried blood spots would suggest (16). In the Maternal Lifestyle Study investigating prenatal cocaine exposure, 38% of mothers who denied use had neonates with positive meconium assays for cocaine and/or opiates (113). In a study of 5,231 mothers attending antenatal clinics in the Cape Town region of South Africa, the prevalence of self-reports of illicit drug use was 3.6% but random urinalyses of 600 mothers showed 8.8% of this subsample met standardized cut-off criteria to test positive for at least one illicit drug and 8.1% for methamphetamine (114).

Women with substance use disorders are less likely to enter treatment than men (14, 15). This is often due to a lack of services tailored to accommodate mothers and their children or the absence of specialist services, particularly in rural regions (115, 116). Furthermore, most women in need of treatment feel they don't need it (14, 15). This suggests a need for intervention approaches that are brief enough to be acceptable even to those who are unwilling to engage in formal treatment but are also accessible and overcome the barriers of stigma and reticence reported by clinicians involved in maternity care. Emerging evidence suggests that brief interventions delivered with the use of computer-based or mHealth technology may provide a more equitable way to overcome the barriers identified by clinicians and the perceived risks identified by women reporting alcohol and drug use during pregnancy.

### Evidence for Screening, Brief Intervention, or Referral to Treatment

Central to the 2015 United Nations Sustainable Development Goals are reproductive and child health and the need to reduce maternal and infant morbidity and mortality, strengthen the prevention and treatment of substance abuse and ensure universal health care and information (117). Consistent with these goals, in 2016, the World Health Organization (WHO) has recommended universal screening for alcohol and other drug use at every antenatal visit in their “WHO recommendations for antenatal care for a positive pregnancy experience” (Table 1) (118). Also, in 2016, WHO recommended the use of SBIRT, a public health framework, to deliver early intervention and treatment services for women with substance use disorders. SBIRT has also been recommended by the Substance Abuse and Mental Health Association (119), the US Preventive Services Task Force (120), and the American College of Obstetricians and Gynecologists (121). Finally, the use of brief interventions and screening is listed as one of 10 core recommendations in the Centers for Disease Control report on preventing alcohol-exposed pregnancies (122).

**Table 1:** United Nations sustainable developmental goals for reproductive and child health.

3.1	By 2030, reduce the global maternal mortality ratio to <70 per 1,000,000 live births.
3.2	By 2030, end preventable deaths of newborns and children under 5-years of age, with all countries aiming to reduce neonatal mortality to at least as low as 12 per 1,000 live births and under 5-mortality to at least as low as 25 per 1,000 live births.
3.5	Strengthen the prevention and treatment of substance abuse, including narcotic drug abuse and the harmful use of alcohol.
3.7	By 2030, ensure universal access to sexual and reproductive health care services, including for family planning, information and education, and the integration of reproductive health into national strategies and programs.
3.9.d	Strengthen the capacity of all countries, in particular developing countries, for early warning, risk reduction and management of national and global health risks.

*United Nations Sustainable Development Group (117)*.

**Figure 1:**
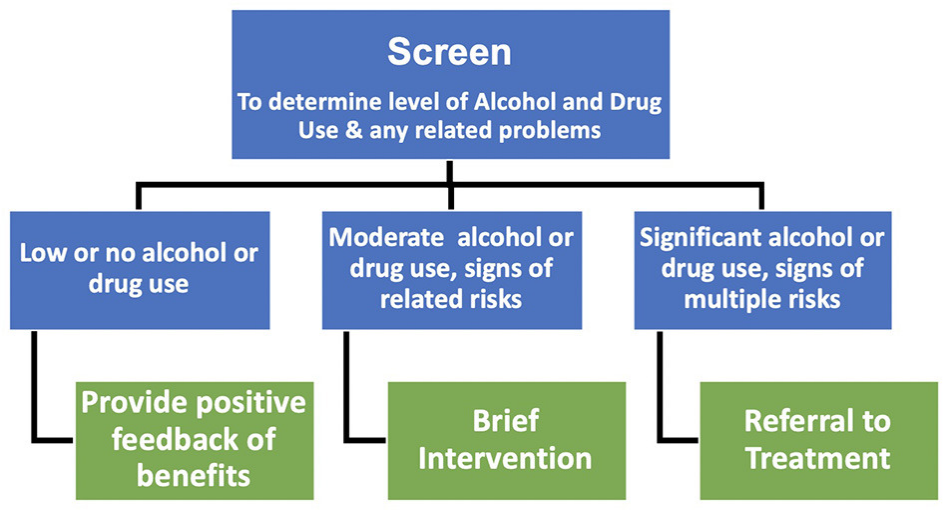
Screening, Brief Intervention and Referral to Treatment (SBIRT).

SBIRT has been applied to the management of alcohol and drug use, smoking cessation, anxiety, and depression (119). The potential of SBIRT is also recognized across the youth health sector as a way to engage young people into treatment to reduce substance use (123). In the present report the focus is the prevention and treatment of harmful alcohol and illicit drug use during pregnancy (Figure 1). In this capacity, Screen refers to screening for the level of alcohol and illicit recreational drug use and to provide positive feedback to women who report they are abstaining or using low levels of alcohol or drugs (see Appendix I for a list of standardized screeners specific to pregnancy). Brief Intervention refers to providing brief motivational messages about the benefits of reducing or abstaining from use and provides the evidence for the benefits of reducing alcohol and drug use during pregnancy for the mother and her baby. Referral to Treatment refers to providing referrals to specialist services that treat alcohol and substance misuse when women report they are continuing to use alcohol and common illicit recreational drugs that meet the criteria on standardized measures for problem use.

## Review of Current Evidence for the Effectiveness of SBIRT Delivered Through mHEALTH Technologies

Despite the initial recommendations and promise of SBIRT, its application has been hampered in the clinical setting in the US, Europe, Australia, and Brazil (124). Challenges include time constraints, lack of buy-in by clinicians, privacy issues, lack of adequate training and lack of intra- and interorganizational communication and collaboration. Additional challenges to implementation of SBIRT that prenatal care providers identified included lack of rapport between providers and women presenting for an initial prenatal consultation; misperception that there is a low prevalence of alcohol and other drug use during pregnancy; perception that women will under report their use and therefore screening is of little use; and providers worrying they will create guilt or anxiety in their patients (20, 41, 107).

With the advancement of mobile technologies and the near-universal access to these technologies, has come the opportunity to provide alcohol and drug services to a typically hard to reach and underserved group of women. Technology in the form of apps and social media is increasingly used to facilitate SBIRT with encouraging preliminary evidence particularly among studies of adolescent girls and women (107, 125, 126). The emerging evidence for the potential effectiveness and usability of SBIRT delivered through mHealth technology (sometimes referred to as e-SBI) to reduce alcohol and illicit substance use in women during pregnancy or the early post-partum period has largely come from reports by one research group (32–40, 127–130). Four of these reports are randomized controlled trials (RCTs) directed toward screening and brief interventions for substance misuse (33, 39, 129, 131), two were small preliminary RCTs of a computer-delivered screening and brief intervention (e-SBI) (34, 36), and a further RCT of e-SBI in a larger sample of post-partum women at risk of problem drinking (Table 2) (40). The balance of the reports from this group have provided important information as to the content, feasibility, usability and cost-effectiveness of brief interventions delivered by clinicians or electronically e-SBIRT (32, 35, 37, 38, 127, 130). These studies have been selected for review as they provide preliminary evidence that technology can be used to deliver SBIRT directly to women during pregnancy or post-partum. Using this approach removes the barriers for women reluctant to report their alcohol and drug use and women in rural locations, with limited resources or transport or other barriers to accessing alcohol and drug services. For a more comprehensive overview of research using technology to address smoking alcohol and substance use and related problems, see Appendix II.

**Table 2:** Evidence of selected RCTs investigating technology delivered brief motivational interventions, SBIRT and e-SBIRT or e-SBI.

**References**	**Sample**	**Target behavior**	**Design and intervention**	**Outcome**
Ondersma et al. (39)	*N* = 107 post-partum low-income women (97% African American) from urban obstetric hospital, inclusion based on any illicit drug use in month prior to pregnancy, US	Reduction in illicit drug use, measured with self-report (ASSIST) and urinalysis	Intervention was 45-min drug use assessment followed by one 20 min interactive brief motivational session delivered on touch-screen laptop (e-SBI) and two follow-up brochures mailed at 4 and 9 weeks, controls only received 45-min assessment	Follow-up at 4 months showed a significant reduction in frequency of use of amphetamine, opiates, and cocaine overtime for the intervention group, but not cannabis–trends for cannabis use and proportion of participants using drugs at follow-up by self-report and/or urinalysis showed small to moderate trends that supported intervention group
Ondersma et al. (129)	*N* = 143 post-partum low-income women (91% African American) from 3 urban hospitals, inclusion based on any illicit drug use in month prior to pregnancy, US	Reduction in illicit drug use, measured with ASSIST at baseline TLFB at follow-up and analysis of 1.5″ hair sample	Both intervention and control group were given 30 min assessment of drug use, intervention group received 20 min e-SBI, controls received time-control condition that included brief video clips and questions about preferences in music and TV	Follow-up at 3 and 6 months showed a significant difference in past 7 days abstinence at 3 months (26% intervention vs. 10% controls), at 6 months no significant difference but similar trend (14 vs. 10%), intervention group reported fewer days of drug in past 90 days (median = 26 vs. 51, *p* = 0.058 at 3 months and 32 vs. 77, *p* = 0.21), hair toxicology results indicated negative results for 29% for intervention vs. 8% for control, *p* = 0.018
Martino et al. (33)	*N* = 80 pregnant *N* = 359 non-pregnant, 67% African American, 13% Caucasian, 14% Hispanic, multiple ethnic groups in two hospital-based health clinics, inclusion based on postive score on ASSIST, US	Reduction of alcohol, misuse of prescription drugs and illicit drugs, drug use identified by ASSIST	Compared, SBIRT delivered in person vs. e-SBIRT delivered on A-CASI vs. Enhanced Usual Care (EUC), and compared usability between SBIRT and e-SBIRT	Follow-up at 1, 3, and 6 months showed a significant decline in days of primary substance use in both SBIRT and e-SBIRT vs. EUC, no difference between groups in treatment utilization, no difference in satisfaction of intervention, between e-SBIRT and SBIRT no differences in referral to treatment
**Alcohol only studies**				
Tzilos et al. (36)	*N* = 50 pregnant low-income women (82% African American), from a urban prenatal clinic who screened positive for risky drinking on the T-ACE, US	Feasibility to detect at-risk drinking and useability of computer delivered, e-SBI intervention, effect size estimate of alcohol use (TLFB), birth outcomes	Intervention was 15-20 min computer-delivered brief intervention (e-SBI) or assessment-only	Follow-up at 30 days revealed high ratings of ease of use and helpfulness. Significant decrease in alcohol use at follow-up but no differences between groups, but intervention mothers had infants that weighed significantly more at birth
Ondersma et al. (34)	*N* = 48 pregnant women (81% African American) from an urban prenatal clinic who screened positive for risky drinking on T-ACE and 1 item from NIAAA, US	Feasibility and acceptability of e-SBI and estimated effect size estimates of intervention on 90-day alcohol abstinent	Pilot RCT, intervention was 15–20 min e-SBI plus three tailored mailings on FASD, controls received interactive discussion on nutrition	High ratings of feasibility and helpfulness (4.7–5.0 on 5 point scale). Follow-up at childbirth revealed medium-size intervention effects on 90-day period prevalence abstinence and intervention effects favoring a health pregnancy variable that included live birth, normal birth weight, and no NICU stay
Ondersma et al. (40)	*N* = 123 post-partum low income women, (87% African American), self-report of drinking 4 or more drinks at a time twice/month and scoring 2 or > on T-ACE, US	Reduction in 7-day alcohol use including fewer days of alcohol use, heavyuse and higher number of days 7-day abstinence post-discharge at 3 and 6 months	Multi-site study, 30-min assessment of alcohol and other drug use obtained *via* the ASSIST followed by 20 min e-SBI, controls received assessment only but to mirror intervention control asked questions about music and TV preferences.	Follow-up at 3 and 6 months showed no clear effectiveness of a single-session, computer-delivered brief intervention at 3 or 6 month follow-up, however, a higher proportion of those receiving e-SBI had more days abstinent than controls at 3 months (37 vs. 26%) and 6 months (42 vs. 38%) findings at 3-month follow-up suggested that greater power might confirm transient effects of e-SBI

*MES, Motivational Enhancement System; SBIRT, Screening, Brief Intervention, or Referral to Treatment; e-SBI or e-SBIRT, electronically delivered Screening, Brief Intervention or SBIRT; A-CASI, audio-enabled, computer-assisted self-interview; ASSIST, Alcohol, Smoking, and Substance Involvement Screening Test; T-ACE, Tolerance, Annoyed, Cut down, Eye-opener alcohol screener; TLFB, TimeLine FollowBack alcohol and drug use interview to identify past use over designated period of time (e.g., past 30 days); NIAAA, National Institute on Alcohol Abuse and Alcoholism Task Force on Recommended Alcohol Questions*.

The first RCT from this group was delivered to post-partum women after the birth of their baby and before discharge from the hospital in their private room (39). The intention of this study was to determine whether a single computer-based intervention session combined with follow-up mailings would decrease new mothers' drug use in the 4 months after the birth of their babies. The sample (*N* = 107) was predominantly African American (97%), low-income women. Of this group, 55 women were randomized to receive a 45-min assessment of their illicit drug use with the WHO Alcohol, Smoking, and Substance Involvement Screening Test (ASSIST) and a single brief intervention. These were delivered on a lap-top fitted with a touch screen. The control group (*N* = 52) received the same assessment of their illicit drug use but no further information. The ASSIST evaluates frequency of use as well as consequences of use for all categories of substances separately (132). At baseline the ASSIST was used to inquire about illicit drug use in the 3 months prior to pregnancy. At 4 months follow-up, the ASSIST was again administered and participants were asked to report their illicit drug use in the previous 3 months, and a urinalysis was conducted for methamphetamine, cocaine, marijuana, opiates, and benzodiazepines. The primary outcome was illicit drug use as measured by urinalysis and the ASSIST self-report at 4 months. The intervention was 20 min in length and delivered by software that featured a three-dimensional animated narrator who read all the material and consisted of three components based on motivational interviewing that focused on the following, (1) feedback regarding negative consequences of self-reported drug use and readiness to change, and the participants drug use in comparison to other women; (2) a pros and cons section that asked participants to indicate positive and negative aspects of drug use from their own perspective; and (3) a summary of the interview and questions about the participant's interest in change, followed by goal setting around ongoing drug use. The results of the self-report data from the ASSIST indicated significant between-group differences in changes in drug use over time that favored the intervention group. This difference was significant for frequency of drug use averaged across all substances (*p* = 0.042), for all illicit drugs with the exception of marijuana *p* = 0.032, but not for marijuana alone *p* = 0.202. Although, point-prevalence analysis of drug use at follow-up did not show significant group differences in drug use, trends again favored the intervention group with effect sizes in the small to moderate range (odds ratios 1.4–4.7). Finally, women who use drugs typically quit or cut down during pregnancy, but many, return to pre-pregnancy use (133). Consistent with this finding, women in the control group returned to using drugs except cannabis at pre-pregnancy rates. Women in the intervention group reported less drug use than in pre-pregnancy suggesting they may have been able to maintain pregnancy-related lower rates in drug use. A thorough search of the literature revealed this study was the first evidence that screening for drug use and a brief intervention delivered completely by technology (e-SBI) could reduce illicit substance use in early post-partum women. However, generalizability of these results is limited due to the small predominantly African American urban sample and an attrition of 29%. A further limitation of the reported findings is the lack of consideration in the analyses as to the influence of the two mailings that were sent out at 4 and 9 weeks postnatally that provided general (non-tailored) information about infant and maternal health and encouraged behavior change for smoking and other behaviors in general.

Replication of the above results with three advancements in study design were carried out in a larger sample (*N* = 143) of post-partum women in a multi-site RCT that included three urban hospitals (129) Added to the protocol for this study were the following: (1) a longer follow-up (6 months) to test the stability of any observed effects of the intervention beyond the 3 month follow-up; (2) the use of the timeline follow-back interview delivered electronically (TLFB) to examine whether there were fewer days of drug use in the past week and past 90 days preceding each follow-up (134); and (3) the deletion of the two motivational mailings to eliminate the potential influence of these on any observed change in drug use. Participants provided urine samples at each follow-up and provided a 1.5-inch hair sample at the 6-month visit which provided an ~90-day window of drug use detection. The primary outcome was 7-day point-prevalence of abstinence at each follow-up point using the results of the TLFB interview and a negative urine toxicology test at 3 months and negative urine plus hair analysis at 6 months. Abstinence was defined as a self-report denying any drug use in the 7 days prior to the follow-up assessment and a urine sample that was negative for cocaine, amphetamines, marijuana, or opiates. A co-primary outcome was number of substance-using days in the 90 days prior to each follow-up. Attrition at 3 and 6 months was 26.6 and 34%, respectively. Point prevalence analyses and analyses of those remaining in the study at 3 and 6 months showed similar between group differences and strengths of association as analyses where those lost to follow-up were all assumed to have used drugs. Of those remaining in the study at 3 months 37.3% reported abstinence in the past 7 days, compared to 13.7% of controls (*p* = 0.006, OR 3.28, CI 1.3, 8.39) and at 6 months 21.7% reported abstinence compared to 15.6% of controls (*p* = 0.449, OR 1.47, CI 0.53, 4.12). Past 90-day drug use at 3 months (26.5 vs. 51.4 days, *p* = 0.58, *d* = 0.60) and 6 months (31.6 vs. 77.2 days, *p* = 0.207, *d* = 0.59) in the intervention group was less than the control group but differences were not significant. Findings from the hair toxicology results showed negative results for 11 (28.9%) participants in the intervention group and 3 (7.9%, *p* = 0.018, OR 4.8, Logit *d* = 0.86) in the control group. These results provided further support for the efficacy and feasibility of an e-SBI in reducing post-partum drug use and extends the original study (39) by following participants to 6 months using TLFB to measure drug-using days. This study also demonstrated that the e-SBI could be effective on its own without the follow-up mailings. However, it did not find evidence for the persistence of intervention effects at 6 months. As with the previous study, the sample was again predominantly African American (91%) and the ASSIST at baseline indicated the predominant drug used by women in these analyses was marijuana, therefore, these results may not generalize to other patterns of drug use or other ethnic groups.

A further RCT to reduce alcohol and illicit substance use included pregnant and non-pregnant women from two hospital-based reproductive healthcare centers (33). All women were screened with the ASSIST for cigarette smoking, alcohol, illicit drugs or prescription medication. Screening took place in regularly scheduled health visits and was undertaken using an audio-enabled, computer-assisted self-interview (A-CASI) tool. Women who scored between 4 and 26 on the ASSIST (range recommended for brief intervention) were randomized into three groups: electronically delivered SBIRT (e-SBIRT, *N* = 143, 16.8% pregnant), clinician delivered SBIRT (*N* = 145, 18.6% pregnant) and a control condition enhanced usual care (EUC, *N* = 151, 19.2% pregnant). All women in the study received a handout that listed local treatment and self-help services. Both intervention conditions (e-SBIRT and SBIRT) received a single 20-min SBIRT session based on motivational interviewing (39, 127, 135). Enhanced usual care consisted of an educational pamphlet plus existing treatment resources. Assessments were completed at baseline and at 1-, 3-, and 6-months. TLFB interviews and urine samples were used to collect information on a wide range of licit and illicit drug use. The co-primary outcomes were self-reported days of primary substance use per month (28 days), and treatment utilization (substance use treatment and self-help programs), both assessed at 1-, 3-, and 6-months post-randomization. Treatment utilization was verified with treatment providers but use of self-help programs relied on participant self-report. Retention at all follow-up points exceeded 84% and were comparable between groups. Both e-SBIRT and SBIRT significantly reduced days of primary substance use over the follow-up period compared to EUC for both pregnant and non-pregnant women. At 1, 3, and 6 months, days reduced substance use from baseline was 3.4, 7.0, and 7.6 (e-SBIRT), 3.0, 6.2 and 6.6 (SBIRT), and 1.6, 4.0, and 5.6 (EUC), respectively. Neither e-SBIRT or SBIRT influenced treatment utilization, even after adjustment for drug use severity. Of those women who reported accessing treatment (27.6%) approximately half of the services they sought were for smoking cessation. This is consistent with the findings that most of the participants met diagnostic criteria for nicotine disorder (56.4%) compared to criteria for cannabis (33.7%), alcohol (27.7%), and an illicit drug use disorder (20.2%). However, as the authors suggest finding a treatment, organizing appointments, and arranging transportation may have been barriers for this low-income population accessing treatment. A subsequent cost-effectiveness study comparing the three methods in this study has found that e-SBIRT compared to EUC and SBIRT is likely to be good value from both the health care provider and the patient's perspective for improving abstinence in women seeking routine care in a reproductive health center who used cigarettes, risky amounts of alcohol, illicit drugs or misused prescription medications (130).

Three trials have focused on using e-SBI specifically to address risky alcohol use in pregnancy or the post-partum period (34, 36, 40). An initial trial of an e-SBI for alcohol use in pregnancy sought to determine the feasibility and acceptability of a computer-delivered brief intervention for alcohol use among women attending a prenatal care clinic (*N* = 50, 82% African American, low income) (36). The secondary aim of this study was to conduct a preliminary effect size estimation of intervention-related changes in alcohol use. Risky alcohol use and inclusion in the study included a T-ACE [Tolerance, Annoyed, Cut down, Eye-Opener score (136)] that met criteria for problem alcohol use, exceeding the National Institute on Alcohol and Alcoholism (NIAAA) “normal” sensible drinking limits before pregnancy (more than seven standard drinks a week or more than two drinks at a time), or reporting drinking at least one time per month during pregnancy. In this trial, 27 women were randomly assigned to receive a single 15–20 min computer-delivered brief intervention at a regular clinic visit that included specific tailored information about FASD (36). Consistent with earlier studies a three-dimensional narrator delivered the intervention on a touch screen laptop and participants listened to the narration through headphones and were then asked questions about the acceptability. The control group were administered a series of questions about television show preferences, viewed a brief series of videos of popular entertainers/shows, and asked questions about their preferences. Follow-up was by phone interview at 1 month using the TLFB to assess alcohol use in both groups. Retention at 1-month follow-up was 96%. Participants rated the brief intervention highly on all measures of satisfaction including ease of use, and respectful and interesting content. Alcohol use decreased significantly within each group, but there was no significant difference between groups in the decrease in alcohol use. However, infants born to mothers in the intervention group weighed significantly more at birth (*M* = 3,189.6, *SD* = 328.0 vs. *M* = 2,965.3, *SD* =387), *d* = 0.62. A number of explanations were proposed for a decline in alcohol use within both groups and the finding of no differences between groups. These included beneficial effects of the intervention coming at a later date than 1-month post-intervention. Also, it was suggested that the effect of asking in-depth questions about alcohol use alone could incur behavior change. Finally, the use of a phone-based interview may have made women reticent about disclosing their alcohol use. For instance, 72% of participants reported drinking at baseline (face-to-face interviews), and only 10% at follow-up phone interviews.

A further pilot randomized trial was designed to address some of the limitations of the above study (34). Included in this trial were tailored enhancements to the video content, the addition of an in-person follow-up, and three tailored mailings post-intervention that extended contact with the participants. The enhanced videos featured a physician providing gain-framed information about alcohol use in pregnancy and a mother providing a testimonial about her decision to avoid the use of alcohol during pregnancy. Multiple versions of these videos were available and designed to address three participant characteristics: quit status, self-efficacy, and frequency of binge drinking. Participants were women (*N* = 48) attending a prenatal clinic who screened positive for alcohol risk on the T-ACE, a single item on the NIAAA about binge drinking in the year before becoming pregnant, and a single item regarding frequency of drinking in the past month. Participants were randomly assigned to the e-SBI plus three separate tailored mailings. Controls received a 20-min interactive intervention focused on nutrition. The primary outcome was feasibility and acceptability of the video enhanced e-SBI. The secondary outcome was the effect estimate of the e-SBI against the control condition for 90-day period-prevalence abstinence obtained using the TLFB after the birth of the baby in the hospital prior to discharge. Participants rated the intervention as easy to use and helpful (4.7–5.0 on a 5.0-point scale). Follow-up evaluation at childbirth by assessors masked to intervention group revealed medium-size intervention effects on 90-day period prevalence abstinence (OR = 3.4, 95% CI 0.5–21.0, *p* = 0.19). Medium size effects (OR = 3.1, 95% CI 0.8–13.8, *p* = 0.09) were also noted on a healthy baby combined variable that included: live birth, normal birth weight, no days in the NICU.

A further RCT investigated the use of e-SBI with a larger sample of post-partum women (*N* = 123) who met criteria on the T-ACE for at risk alcohol use (40). Participants were largely African American women (87%) who delivered their baby at an urban hospital. The intervention was delivered to mothers in their private hospital room prior to discharge post-birth of their baby. As in the previous studies a single 15–20 min, tailored, brief intervention was delivered on a tablet PC by a three-dimensional narrator and listened to by participants on headphones. The ASSIST was used at baseline to identify frequency of use as well as consequences of use and referred at baseline to use in the 3 months prior to pregnancy in order to promote disclosure and establish a baseline more reflective of a drinking pattern when not pregnant. Background demographics, mental illness, current relationships and treatment history. TLFB evaluated alcohol use in the past week and past 90 days. The NIAAA was used to identify quantity-frequency and binge drinking. The hypothesis of this study was that women receiving the intervention would report higher 7-day point-prevalence abstinence from alcohol and fewer days of alcohol use at 3 and 6 months postnatally. Results showed small effect sizes, and no clear differences between groups for 7-day point prevalence at 3 and 6 months for abstinence, number of drinking days in the past 90 days or mean number of days per week. However, of those who completed the study at 3 months, 15 of 41 e-SBI participants (36%) were abstinent vs. 11 of 42 (26.2%) in the control condition and at 6 months this trend again favored the intervention group [17 of 45 (37.8%) e-SBI vs. 17 of 41 (37.8%) controls].

Electronically delivered SBIRT also allows incorporation of text messaging tailored to the responses women make to screening questions (37). The benefit of text messaging is that it can maintain multiple communications with a participant, reach participants in their home environment, and are nearly always opened (99% are opened, 90% within the first 3 min of receipt) (137). Ondersma et al. investigated the feasibility and acceptability of text messaging combined with e-SBI or e-SBI delivered alone on a tablet PC (38). In this study, pregnant women seeking prenatal care services and scoring positive for cannabis use risk were randomly assigned to receive e-SBI (*N* = 15) or e-SBI with text messaging (*N* = 15) or only text messaging (15). Both interventions were delivered using the Computerized Intervention Authoring System (CIAS), an authoring tool designed for creation of e-Health and mHealth interventions. Both brief interventions were designed to be delivered with no involvement of a therapist. The brief intervention was ~20 min long and began with a 4-min video tailored to age, race, quit status, and self-efficacy. This was delivered by a physician that provided accurate information about marijuana use in pregnancy. The balance of the video (~16 min) used an animated narrator similar to previous research from this group to deliver the intervention. Participants in the text messaging group could nominate the frequency of texts per week and time of day they wanted to receive these. Text content included mixed targeted messages about healthy pregnancies (e.g., nutrition), information regarding community resources, and inspirational quotes. Results of this study supported the feasibility and acceptability of the e-SBI and all participants were able to complete the intervention during clinic time and gave it very high ratings. Importantly, nearly half of participants asked for messages more often than once per week, and more than three-quarters chose to continue receiving messages throughout pregnancy—with a median overall receipt of 24 messages.

## Discussion

A recent systematic review of the usability and effectiveness of mHealth technology on supporting health care during pregnancy (37) and a recent meta-analysis of effectiveness of mHealth apps and social media (5) show that prenatal care delivered with the aid of technology has the potential to deliver effective and more equitable prenatal care. Together they show the usability and effectiveness in providing prenatal care to reduce gestational weight gain and manage gestational diabetes and asthma, to promote overall physical health and improve nutrition, and improve mental health and knowledge about pregnancy. However, both argue for more research that include larger studies and more diverse populations.

### Summary of the Evidence for e-SBI or e-SBIRT

The evidence from the studies reviewed in this report, provide emerging evidence for the effectiveness of a public health approach to delivering universal screening and substance use treatment during pregnancy and the post-partum period to a particularly high-risk population with complex needs. Intervening during pregnancy or the early post-partum period has the potential to prevent the prenatal and postnatal effects of parental drug use reviewed earlier in this report. Pregnancy and the birth of a child is a “window of opportunity” where mothers are particularly open to changing their behavior to benefit the health of their child (138). Therefore, treatment delivered at this point also have the potential to be more readily accepted and in turn reduce or eliminate ongoing alcohol or drug use which is likely to benefit both the mother and her child. Delivered electronically, without the aid of a therapist, e-SBIRT or e-SBI has the potential to overcome a number of the barriers common to women who use alcohol and illicit drugs, particularly those who have alcohol or substance use disorders. However, the population under investigation in many of these studies has been predominantly African American and from lower-socioeconomic urban populations in the US where there is a legal mandate to report illicit drug use in certain states. Therefore, women who may benefit most from e-SBI may not have been included in these studies due to fear of potential involvement with child protection services (17) or living in rural areas where there are few resources to treat alcohol or drug use. Finally, the evidence from the reviewed studies focus mainly on alcohol or substance use and haven't included many of the psychiatric and social problems common to maternal drug use, particularly co-morbid psychiatric disorders (72).

Finally, although most of these studies have obtained data on acceptability and usability, there hasn't been an emphasis on asking about other information or content this group of women might find useful. Evidence from a growing body of literature on service integration shows that substance abuse treatment for women is more effective when health, mental health, parenting, vocational, housing and legal issues are addressed along with the substance abuse issues (139, 140). One recent report describes a promising approach to including a wider range of content to support evidence-based prenatal screening, brief intervention, and referral to treatment for risk and protective factors in pregnancy (41). Using qualitative interviews with clinicians and end-users of SBIRT, they used an iterative process that began with including women with a complex history of anxiety, depression, substance use, gestational diabetes overweight, and/or intimate partner violence and clinicians that included midwives, obstetricians and mental health workers involved in the care of this population in the development of the content. After the testing of the app with patients, they conducted interviews with clinicians and provided training to show them how to implement mHealth technology into their practice.

### Considerations in the Design of mHealth Apps

Research in the effectiveness and useability of mHealth to treat women of childbearing age is just beginning. However, given the steep increase in mobile technology and drug use by women worldwide it is an important area of inquiry. A recent study found that 50.7% of pregnant, first-time mothers sought health information online and 22.4% used an mHealth app (141). Women using these apps were more likely to be younger, were more likely to be in their first pregnancy and reported feeling less healthy. They were also more likely to be influenced by the information they received. Therefore, the integrity of the information they receive regarding the impact of drug use during pregnancy and the post-partum period is of significant importance and should be rigorously evaluated. Also, if we want the uptake of mHealth apps by our target audience then co-design of these apps that address the culture, ethnicity and unique needs of the target audience are required (142). Important also for uptake is the useability and feasibility. The studies reviewed here have thoroughly examined the feasibility and usefulness of e-SBI, and future investigations should continue to collect this information along with a measure of behavioral change. Lacking in the literature is evidence around how to protect the security of the information women provide who are using these apps. Therefore, future research should investigate how to protect the security and privacy of the mHealth apps used in healthcare, particularly for this high-risk population of women with complex social, legal and health needs.

## Conclusions

In conclusion, alcohol and substance use during pregnancy is an escalating public health problem. Emerging evidence shows that technology has a part to play in reducing and treating maternal drug use. SBIRT is a proven approach to screening, brief intervention and referral to treatment in a wide range of populations and emerging evidence suggests it could be more equitably delivered to women in pregnancy and the postnatal period using technology. Given the ubiquity of smart phones and mobile devices, e-SBI or e-SBIRT has the potential to be a more equitable and effective approach to treating a particularly high-risk complex population that has previously been hard to reach.

## Author Contributions

TW developed the topic for the review and wrote the first draft of the review and edited subsequent drafts. KS consulted on the topic of mHealth technology for the draft and commented on the draft. SS developed the table of screening instruments for alcohol and substance use screeners included in the Supplementary Material, and commented on the draft. AC searched the literature and identified relevant articles for the review, developed the table of relevant articles providing an overview of brief interventions included in the Supplementary Material, and commented on the draft. All authors contributed to the article and approved the submitted version.

## Conflict of Interest

The authors declare that the research was conducted in the absence of any commercial or financial relationships that could be construed as a potential conflict of interest.
