# Supplementary material for: Evidence for the Effectiveness and Acceptability of e-SBI or e-SBIRT in the Management of Alcohol and Illicit Substance Use in Pregnant and Post-partum Women
Source: Front Psychiatry. 2021 May 5;12:634805. doi: 10.3389/fpsyt.2021.634805 (PMC8131659; doi:10.3389/fpsyt.2021.634805)
Supplement: Supplementary file 1 [file Table_1.DOCX]

Appendix I: Brief screening instruments for alcohol, tobacco and illicit substance use in pregnancy

| **Instrument** | **Description** | **Administration method** | **Substance** | **Validated in pregnancy** | **Risk group(s) identified** |
| --- | --- | --- | --- | --- | --- |
| WHO-ASSIST^1^  V3^2^ | 8-items* | Interviewer | Alcohol, tobacco, substances | No^3^ | Lower, moderate or high risk score for each substance. Aligns with ‘no treatment’, ‘brief intervention’ or ‘referral to treatment’ respectively |
| ASSIST-LITE^4^ | 7-items^§^ | Interviewer, online, app | Alcohol, tobacco, substances | No^5^ | Risk score for each substance |
| NIDA Quick Screen^6^ | 4-item pre-screen | Interviewer | Alcohol, tobacco, substances | Yes^7, 13^ | ‘At-risk’ alcohol or tobacco use cut offs – clinical follow-up. Any substance use – NIDA modified ASSIST administered |
| NIDA modified ASSIST V2.0^6^ | 8-items* – follows positive NIDA Quick Screen | Interviewer | Illicit drug | Cannabis only^7,8^ | Lower, moderate or high risk score for each substance. Aligns with ‘no treatment’, ‘brief intervention’ or ‘referral to treatment’ respectively |
| 4-Ps – Plus^7 #^ | 4-items - modified for use in pregnancy | Paper and pen/computer | Alcohol and general substance use | Yes^10,7^ | Single cut off: Low – high risk identified: ≥1 score = positive screen |
| 5-Ps | 5-item screener | Paper and pencil, computer | Alcohol and substance use | Yes^13^ | Cut-off score 1: Revised version of 4-Ps, added question about peers |
| CRAFFT | 6-item screener | Interview and pen/computer | Substance use | Yes^13^ | Cut-off score 2: Using calendar reference, in adolescents, young women/pregnant. |
| WIDUS^12, 13^ | 6-item screener | Interviewer, computer | Substance use | Yes^13^ | Cut-off score 3: Indirect screener to identify risk in perinatal period by asking about correlates of drug use. |
| SURP-P^11^ | 3-items | Paper and pen/computer | Alcohol and substances | Yes^11,7,13^ | Flexible use: Low risk population, ≥1 score = positive screen; High risk population: ≥ 2 = positive screen. |

ASSIST - Alcohol, Smoking and Substance Involvement Screening Test

NIDA - National Institute on Drug Abuse

WHO - World Health Organisation

4-Ps Plus - Parents, Partner, Past, Pregnancy & 5-Ps Parents, Peers, Partner, Pregnancy, Past

CRAFFT – Car, Relax, Alone, Forget, Friends and Trouble

SURP-P- Substance Use Risk Profile-Pregnancy

WIDUS – Wayne Indirect Drug Use Screener

^#^ licensing fee applies; *each item asks about 10 classes of substances. §items 1-6 ask 2-3 follow-up questions.

Brief screening instruments for alcohol use

| Instrument | Description | Validated in pregnancy | Risk groups identified |
| --- | --- | --- | --- |
| CAGE | 4-items | No | Heavy alcohol use only |
| AUDIT-C | 3-items (adapted from AUDIT) | No | Heavy alcohol use only |
| NET | 3-items | Yes | Heavy alcohol use only |
| T-ACE | 4-items | Yes | Heavy alcohol use only |
| TWEAK | 5- item screener | Yes | Moderate to heavy alcohol use |

CAGE: Cut down, Annoyed by criticism, Guilty about drinking, Eye-opener; AUDIT-C: Alcohol Use Disorders Identification Test – Alcohol consumption questions; NET: Normal drinker, Eye-opener, Tolerance; T-ACE: Tolerance, Annoyed by criticism, Cut down, Eye-opener; TWEAK: Tolerance, Worry about drinking, Eye-opener, Amnesia, K/Cut down.

References

1. WHO ASSIST Working Group. The Alcohol, Smoking and Substance Involvement Screening Test (ASSIST): development, reliability and feasibility. Addiction. 2002;97(9):1183-94. doi: 10.1046/j.1360-0443.2002.00185.x.
2. World Health Organization (WHO) Alcohol, Smoking and Substance Involvement Screening Test (ASSIST), Version 3.0, developed and published by WHO (available at http://www.who.int/substance_abuse/activities/assist_v3_english.pdf). Retrieved 19^th^ October 2020.
3. Hotham E, Ali R, White J, Sullivan T, Robinson J. Investigation of the Alcohol, Smoking, and Substance Involvement Screening Test (the ASSIST) Version 3.0 in Pregnancy. Addict Disord Their Treat 2013, 12(3), 123–135
4. Ali R, Meena S, Eastwood B, Richards I, Marsden J. Ultra-rapid screening for substance-use disorders: the Alcohol, Smoking and Substance Involvement Screening Test (ASSIST-Lite). Drug Alcohol Depend. 2013 Sep 1;132(1-2):352-61. doi: 10.1016/j.drugalcdep.2013.03.001.
5. Hothman, E.D., Ali, R. L., White, J.N. (2016). Analysis of qualitative data from the investigation study in pregnancy of the ASSIST Version 3.0 (the Alcohol, Smoking and Substance Involvement Screening Test). *Midwifery, 34.* 183-197. [doi.org/10.1016/j.midw.2015.11.011](https://doi.org/10.1016/j.midw.2015.11.011)
6. National Institute on Drug Abuse. Resource guide: screening for drug use in general medical settings. Available at: https:// [www.drugabuse.gov/publications/resource-guide-screeningdrug-use-in-general-medical-settings](http://www.drugabuse.gov/publications/resource-guide-screeningdrug-use-in-general-medical-settings). Retrieved 19^th^ October 2020.
7. Coleman-Cowger VH, Oga EA, Peters EN, Trocin KE, Koszowski B, Mark K. Accuracy of Three Screening Tools for Prenatal Substance Use. Obstet Gynecol. 2019 May;133(5):952-961. doi: 10.1097/AOG.0000000000003230.
8. Oga EA, Mark K, Peters EN, Coleman-Cowger VH. Validation of the NIDA-modified ASSIST as a Screening Tool for Prenatal Drug Use in an Urban Setting in the United States. J Addict Med. 2020 Sep/Oct;14(5):423-430. doi: 10.1097/ADM.0000000000000614.

*NOTE Oga full paper unavailable so citation is using abstract only.*

1. Chasnoff IJ, Hung WC. The 4P’s Plus. NTI Publishing: Chicago, 1999.
2. Chasnoff, I.J., Wells, A.M., McGourty, R.F & Bailey, L.K (2007). Validation of the 4P’s Plus^©^ screen for substance use in pregnancy validation of the 4P’s Plus. Journal of Perinatology, 27, 744-748.
3. Yonkers, K. A., Gotman, N., Kershaw, T., Forray, A., Howell, H. B., & Rounsaville, B. J. (2010). Screening for prenatal substance use: development of the Substance Use Risk Profile-Pregnancy scale. Obstet Gynecol, 116(4), 827-833.
4. Ondersma, S.J., Svikis, D.S., LeBreton, J.M., Streiner, D.L., Grekin, E.R., Lam, P.K. et al. (2012). Development and preliminary validation of an indirect sceener for drug use in the perinatal period. Addiction;107: 2099-2106.
5. Ondersma, S.J., Chang, G., Blake-Lamb, T., Gilstad-Hayden, K., Orav, J. et al. (2019). Accuracy of five self-report screening instruments for substance use in pregnancy. Addiction; 114: 1683-1693.
