# Supplementary material for: Evidence for the Effectiveness and Acceptability of e-SBI or e-SBIRT in the Management of Alcohol and Illicit Substance Use in Pregnant and Post-partum Women
Source: Front Psychiatry. 2021 May 5;12:634805. doi: 10.3389/fpsyt.2021.634805 (PMC8131659; doi:10.3389/fpsyt.2021.634805)
Supplement: Supplementary file 2 [file Table_2.docx]

**Appendix II.** Overview of technology based interventions for alcohol, tobacco, substance abuse, intimate partner violence and multiple risks

Alcohol and Substance Use

| **Study** | **Study Design** | **Intervention** | **Participants** | **Outcome Measures** | **Outcome** |
| --- | --- | --- | --- | --- | --- |
| Loree, Yonkers, Ondersma et al. 2019 | RCT  Recruitment from 2 Urban academic hospital based health clinics (US: Detroit). | Single session (20 min) SBIRT. Interactive guidance through intervention based on motivational Interviewing  In person delivery (SBIRT) vs electronic delivery (e-SBIRT) vs enhanced usual care (EUC). | Pregnant (N = 80) and non-pregnant (N = 359) women who scored positive on WHO ASSIST substance use screener.  African American (67%), Caucasian American (12%), Hispanic (15%), other (6%)  Included in primary analysis (SBIRT = 145, e-SBIRT = 143, EUC = 151). | Satisfaction: SR survey  Alliance: SR survey  Substance Use: SR total days of use. | High scores for satisfaction and alliance for both in person and electronic delivery.  No association between intervention type/ satisfaction/ alliance associated with substance use. |
| Ondersma, Beatty, Puder et al. 2019 | RCT  Recruitment from 3 Urban academic hospital based health clinics (US: Detroit). | Single session (20 min) Electronic Brief intervention (e-SBI), tailored and interactive with animated narrator guidance.  Text messaging using participant determined frequency (weekly, 2x per week, 3x per week) and time of day (morning, afternoon, evening, night time).  (Developed and refined from Gray et al 2017). | Pregnant women who reported using marijuana in the month before pregnancy.  Text (N = 15)  e-SBIRT (N = 15)  Text + e-SBIRT (N = 15)  African American (84%), Hispanic (2%), Other (14%). | Satisfaction: SR survey  Retention of text messaging program. | High scores for acceptability for e-SBI and some aspects (ease of understanding, respectfulness, and helpfulness) of texting intervention.  Combination of e-SBI and Text messaging showed increased retention. |
| Martino, Ondersma, Forray et al. 2018 | RCT  Recruitment from 2 Urban academic hospital based health clinics (US: Detroit). | Single session (20 min) SBIRT*. Interactive guidance through intervention based on motivational Interviewing  In person delivery (SBIRT) vs electronic delivery (e-SBIRT) vs enhanced usual care (EUC). | Pregnant (N = 80) and non-pregnant (N = 359) women who scored positive on WHO ASSIST substance use screener.  African American (67%), White (12%), Hispanic (15%), other (6%)  Included in primary analysis (SBIRT = 145, e-SBIRT = 143, EUC = 151). | Substance use: Self-reported days of primary substance use per month.  Treatment utilisation (substance use treatment and self-help programs).  7 time intervals | Significant decline in substance use for SBIRT and e-SBIRT.  No difference between SBIRT and e-SBIRT  More modest declines for EUC.  Neither SBIRT nor e-SBIRT resulted in more treatment utilisation than EUC. |
| Gray, Beatty, Svikis et al. 2017 | Pilot intervention study  Urban Prenatal Clinic (US: Detroit). | Single session electronic brief intervention with three dimensional narrator guidance and follow up text messaging plan.  Participants reviewed the intervention materials and reviewed the list of text messages. | Pregnant women who reported marijuana use in month before pregnancy and recruited from prenatal care visit. Patients (N = 10). Medical staff volunteers from the same prenatal clinic.  Provider-participant (N = 12). | Acceptability: SR Survey.  Patient-participant feedback interview.  Provider-participant focus group interview. | Patient- participants  High ratings for acceptability.  5/10 reported preference of e-medicine vs doctor intervention.  9/10 reported e-medicine program increased likelihood of decreasing marijuana use.  Provider-participants  Endorsed the program, reported, information was both relevant and important. |
| Ondersma, Svikis, Thacker et al. 2016 | RCT  Multisite parallel group recruited from urban hospitals (US: Detroit).  . | Single session (20 min) electronic screening and brief intervention (e-SBI) for alcohol use among postpartum women. | Postpartum, low-income women who met criteria for unhealthy alcohol use (N = 123).  African American (87%), white (4%), multiracial (4%), other (5%) (Time Control Condition =61, Intervention = 61). | Satisfaction: SR survey  Alcohol Use:  Alcohol use days  Mean drinks per week  Binge episodes per week. | e-SBI was moderately well received. No group differences between e-SBI and Control were found. |
| Ondersma, Beatty, Svikis et al. 2015 | Pilot Randomised Trial.  Recruitment from urban prenatal care clinic (US: Detroit). | 20 min interactive session. Tailored to consumption change since becoming pregnant and goals.  Based on prior trial (Tzilos et al., 2011). | Pregnant women who screened positive using the T-ACE and SR risky drinking for alcohol risk (e-SBI = 24, Control = 24)  African American (81%), Non-African American (19%) | Feasibility: Ease of recruitment by clinic staff and proportion who were able to complete e-SBI.  Acceptability: SR survey  Substance Use: 90-day abstinence at delivery.  Infant Health: birth weight and no admission to NICU.  Treatment: sought services for any level of alcohol use. | Feasibility reported as high.  High scores for acceptability and perceived efficacy of e-SBI.  Moderate non-significant effect on 90-day abstinence and Infant Health. Only 1 participant sought treatment. |
| Pollick, Beatty, Sokol et al. 2015 | Pilot intervention at Urban prenatal care clinic (US: Detroit). | Single session (20 min) C-BIAP with 3-dimensional animated narrator. Focused on relapse prevention with women who report already abstaining. | Pregnant women who screened positive using the T-ACE.  N= 18, African American (100%). | Acceptability: SR survey.  Qualitative information to elicit overall impression of the software. | High scores of acceptability from SR survey.  Qualitative information suggested:  Decreased relevance as participants already knew information presented.  Broad acceptability and helpfulness of the software.  Many thought the intervention had influenced/reassured their decisions about drinking during pregnancy.  Suggestions included more detail and information on cannabis and cigarettes. |
| Ondersma, Svikis, Schuster et al. 2007 | RCT  Recruited from Urban Obstetric Hospital (US: Detroit).  . | Single session (20 min) computer-based motivational intervention with 2 nontailored mailings and voucher reinforcement of attendance of initial treatment session. | Postpartum women who reported any illicit drug use in the month before pregnancy.  Control group: Assessment only (N=52).  Intervention group: Assessment + Intervention (N = 55).  African American (97%) | ASSIST – baseline completed at birth but measured drug use in the 3 months preceding pregnancy.  Positive drug use 4 months post baseline: urines testing and ASSIST.  Measured Marijuana alone and combined other drug use. | At follow up frequency of other drug use increased for control group. Other drug use intervention showed a significant decrease at follow-up. Decreased use in the marijuana intervention group did not reach significance.  No group differences were found for dichotomous drug use data. |
| Tzilos, Sokol, & Ondersma 2011 | Pilot Randomised Trial.  Urban prenatal care clinic (US: Detroit). | Single session (15-20 min) interactive intervention.  Tailored to current drinking status and interest in abstinence. | Pregnant women who screened positive using the T-ACE, exceeding NIAAA “normal” sensible drinking limits before pregnancy, and SR risky drinking for alcohol risk (Intervention = 27, Control = 23)  African American (82%), Caucasian (16%), Hispanic (4%). | Feasibility: Ease of recruitment by clinic staff and proportion who were able to complete e-SBI.  Acceptability: SR survey.  Infant Health: birth weight, gestational age, head circumference.  Self-recall alcohol use over the past month suing Timeline follow-back. | Good Feasibility.  High scores of Acceptability.  Alcohol use significantly decreased in both Interventional and Control Group. No group differences detected.  No group difference detected for head circumference and gestational age. Birth weight was significantly higher in Intervention group. |
| Ondersma, Chase, Svikis et al. 2005 | Study recruitment from three urban hospital clinics (general obstetric, substance use treatment facility, methadone maintenance program) (US: Detroit). | Motivations Enhancement System (MES) . Assessment and intervention using a computer based interactive motivational interviewing session. | Postpartum women who reported illicit drug use in the month before becoming pregnant. Study 1: Assess Feasibly of Intervention (Motivational Enhancement System; MES) (N= 47).  Study 2: Assess Intervention associated fluctuations in state motivation (N = 40).  Study 3:Pilot clinical trial of MES. (Assessment = 15, Assessment + intervention = 15)  African American (97%), Non-African American (3%). | Study 1: SR feasibility/acceptability instrument alongside qualitative information  Study 2: SR state motivation survey.  Study 3: Drug Use screener, ASSIST, SR Motivation to change measure, review of treatment services. | Women rated intervention as acceptable and easy to use.  Intervention associated with significant increases in state motivation.  No significant differences between intervention and control group on substance use variables. |

- RCT: Randomized Controlled Trial.
- SBIRT: Screening, Brief Intervention and Referral to Treatment.
- SR: Self-report.
- NICU: Neonatal Intensive Care Unit.
- C-BIAP: Computerized Brief Intervention for Alcohol Use in Pregnancy.
- ASSIST: World health Organisation (WHO) Alcohol, Smoking and Substance Involvement Screening.
- IPV: Intimate Partner Violence.

**Tobacco**

| **Study** | **Study Design** | **Intervention** | **Participants** | **Outcome Measures** | **Outcome** |
| --- | --- | --- | --- | --- | --- |
| Abroms, Chiang, Macherelli et al. 2017 | Recruitment from 11 obstetrics-gynecology clinics (US: Washington DC) | SmokefreeMOM, a national smoking cessation text-messaging program for pregnant smokers | Pregnant women who were smoking or had smoked in past 2 weeks..  SmokefreeMOM (n=55)  Control text message quitline referral (n=44)  White (56%), African American (40%), Other (4%) | Participants surveyed by phone at baseline and at 1 month and 3 months after enrolment | SmokefreeMOM rated highly for helpfulness at 3 months, and frequency of messaging at 1 months and 3 month.  Majority of participants read all text messages and few discontinued the program.  No significant between group difference on use of treatment resources or smoking outcomes. |
| Naughton, Cooper, Forster et al. 2017 | RCT  Study recruitment from Antenatal clinics (UK) | Usual Care (UC) smoking cessation leaflet  MiQuit - Smoking cessation support delivered by Short message service (SMS) | Pregnant women who smoked at least one cigarette in pregnancy (>5 pre-pregnancy)  MiQuit (N = 203)  Usual Care (N = 204) | Seven smoking outcomes, including  validated continuous abstinence from 4weeks post-randomization until 36 weeks gestation and biochemically validated. | Some evidence that text-messaging programme may increase cessation rates, but not conclusive. |
| Abroms, Johnson, Heminger et al. 2015 | Recruitment via text message to pregnant women (US: Pennsylvania, Maryland, West Virginia, Nth Carolina, Kentucky Tennessee) | Quit4bay text messaging program based on social cognitive theory. | Enrolled (N = 20)  Completed 2 week follow-up  (N = 16)  Completed 4-week follow up  (N = 13).  White (65%), African American (25%), other ethnicity (10%) | Baseline:  Survey gathering text messaging habits, smoking behaviours, beliefs, motivation for quitting.  Attitude-Social Influence-Efficacy Model (ASE).  2 & 4-week interviews gathering information on smoking behaviours, beliefs and perceptions of Quit4baby.  Satisfaction: SR Survey. | High scores for helpfulness and being personal.  Participants rated highly the strategies and skills offered by Quit4baby.  Improvement ideas included increasing message dose and making the program more interactive. |
| Harris & Reynolds 2015 | US: Rural Appalachian | Web-based contingency management program (CM)  Phone-delivered cessation counselling program (Smoking Cessation for Healthy Births [SCHB]) | Community sample of pregnant women smoking >2 cigarettes per day.  CM (N= 7)  SCHB (N=10)  White (N = 15), Other (N = 2) | Modified Fagerstrom Test for Nicotine  Dependence (mFTND)  Stage of Change Ladder (SCL)  Timeline follow-back calendar for cigarette per day during past 14 days, occurring on a monthly basis beginning one month after enrolment and continued until the end of pregnancy. | CM participants attained abstinence more rapidly than SCHB.  SCHB experienced less relapse to smoking, and a greater percentage of these participants reduced their smoking by at least 50%. |
| Herbec, Brown, Tombor et al. 2014 | RCT  Community recruitment through online advertisement place on NHS Smokefree website  (UK) | MumsQuit internet-based interactive, personalised, structured quit plan with 4 weeks of pre-quit date support and 4-weeks of post-quit date support.  Control intervention one-page non-personalised website that contained standard advice. | Pregnant women who are smoking daily and want to stop smoking.  MumsQuit (N = 99)  Brief-advice control website (N = 101)  White (93%) | Primary outcome was self-reported 4-week continuous abstinence at 8- week follow-up.  Secondary outcomes included automatically collected data on quantifiable website usage. | MumsQuit participants logged in significantly more often, viewed more pages and spent longer time browsing the website.  Small differences between intervention with MumsQuit MumsQuit demonstrating slightly more abstinence than control intervention. |
| Pollak, Lyna, Bilheimer et al. 2013 | Pilot Study  Recruitment from prenatal clinics (US: North Carolina) | Text (SMS) support messages.  SMS support messages plus Scheduled Gradual Reduction (SGR). | Pregnant women who have smoked ≥ 5 cigarettes per day in the prior 7 days. (N = 31) | SR follow-up survey  to assess the usability  and acceptability of the support and SGR text messages 6 weeks post baseline.  Biochemically  validated 7-day point prevalence abstinence. | High scores of feasibility and acceptability.  SGR participants had higher rated of biochemically validated 7-day point prevalence at the end of pregnancy and showed greater reduction in smoking. However, no effect sizes reported. |
| Naughton, Prevost, Gilbert et al. 2012 | RCT  Study recruitment from seven NHS Trusts  (UK: South, South-East and north-East of England). | MiQuit Intervention based on Social Cognitive Change theory, Perspectives on Change Model and Elaboration Likelihood Model.  Participants given 4 page leaflet and text messages tailored to smoking status.  Participants able to request “Instant response” supportive texts.  Control group received Pamphlet trial and assessment but no intervention texts. | Pregnant women who smoked ≥7 cigarettes per week.  Allocated MiQuit (N= 102)  Allocated to Control (N = 105)  100% White participants. | Smoking outcome  SR 7-day point prevalence abstinence at 3 month follow-up and measure of salivary cotinine.  Self-efficacy, harm beliefs, and motivation were measured using SR survey.  Acceptability and usefulness rated by SR and interview. | High scores of feasibility and few participants chose to discontinue.  MiQuit participants were more likely to set a quit date, reported higher levels of self-efficacy and determination to quit.  No statistical difference of self-report abstinence at 3 month follow up. |
| Ondersma, Svikis, Lam et al. 2012 | RCT  Study recruitment from four urban prenatal hospital clinics (US: Detroit). | Computer-delivered 5As-based brief intervention (CD-5As).  Computer-assisted, simplified, and low-intensity contingency management (CM-Lite)  CD5As + CM-Lite  Treatment as usual (TAU) | Pregnant women reporting smoking in the last week.  CD-5As (N = 26)  CM-Lite (N= 28)  CD5As + CM-Lite (N =30)  TAU (N = 26)  African American (82%) | Satisfaction: SR survey  Motivation: DR Survey  Both surveys completed before after intervention.  Baseline dependence   - Fagerstrom Test for Nicotine Dependence (FTND) - K6 measure of emotional distress   10 week follow up.   - 7 day point prevalence abstinence via timeline follow back calendar and test of expired carbon monoxide (CO) levels. - Urine analysis to detect urinary cotinine - sought help for smoking from physician or Quitline. | High scores of satisfaction, acceptability, and motivation for CD-5As.    CD-5As led to increased abstinence as measured by cotinine but not for CO-confirmed 7-day point prevalence.  CD-5As participants were more likely to seek support from a health professional about their smoking.  Lower intensity participant-initiated CM did not influence smoking during pregnancy. |
| Lawrence, Aveyard, Evans et al. 2003 | RCT  Study recruitment from antenatal clinics in General Practice.  (UK: West midlands) | Standard Care (SC) – standard smoking advice given by midwives.  Transtheoretical (stages of change) model (TTM based self-help manuals)  TTM plus interactive and personalised computer program. (CTTM) | Pregnant women who smoked at booking their first antenatal appointment.  Recruited  SC (N = 289)  TTM (N = 305)  CTTM (N = 324)  White (89%), Black (2%), Other (9%) | Biochemically confirmed smoking cessation for 10 weeks previously, and point prevalence abstinence, both measured at 30 weeks of pregnancy and 10 days after delivery. | Small significant increase in quitting in the combined intervention arms compared with the controls. |
| Cinciripini McClure, Wetter et al. 2000 | Recruited from community using, radio, newspaper & television, subscriber newsletters.  (US: Houston) | Usual Care received the VIPS self-help quit calendar and cessation tip guide.  Usual Care plus video also received video program consisting of six 25-30mins videotapes. | Pregnant women who smoke >3 cigarettes per day.  UC (N = 40)  UCV (N = 42)  Non-white (19.9%) | Women were asked to set a quit date and measures of abstinence were obtained 2-3 days after quit date, 4-5 weeks after quit date and 1 month postpartum.  SR ratings of helpfulness, informative and relevance. | No significance between group difference in abstinence or attrition was found.  High scores for helpfulness, informative and relevance for UCV although many participants did not complete watching all videos. |
| Ershoff, Quinn, Boyd et al. 1999 | RCT  Prospective recruitment from managed care organisation  (US: Southern California) | Three intervention groups   1. Self-help booklet tailored to pregnant smokers 2. Booklet plus access to computerised interactive telephone cessation program (UVR). 3. Booklet plus proactive telephone counselling (MI) | Pregnant women who smoked in the 7 days prior to interview and had a prepregancy smoking rate of >7 per week.  Booklet only (N = 111)  IVR (N= 120)  MI (101)  White (61%), African American (15%), Hispanic (16%) | Confirmed abstinence measured by level of cotinine in urine samples  obtained during a routine prenatal visit at approximately 34 week gestation.  Follow-up interview at the 32^nd^ week of pregnancy. | No significant differences found between intervention groups. |

**Intimate Partner Violence**

| **Study** | **Study Design** | **Intervention** | **Participants** | **Outcome Measures** | **Outcome** |
| --- | --- | --- | --- | --- | --- |
| Garnweidner-Holme, Henriksen, Flaathen et al. 2020 | Qualitative study of midwife attitudes.  (Norway: Oslo) | Safe pregnancy study – Tablet intervention to prevent IPV, which included questions about IPV and a video.  Intervention materials have been translated into Norwegian, English, Urdu, and Somali. | Nine midwives from a mother and child health centre who recruited participants for an RCT to test a video regarding IPV during prenatal care | Thematic analysis using semi-structured interview guide. | Tablet intervention:  - was an appropriate supplement to prenatal care.  - was a good solution to ensure every woman get the same information.  - was to be useful if it was also followed with face-to-face intervention.  - had some difficulty when recruiting women from different ethnic backgrounds and recruitment was time consuming. |
| Flaathen, Lukasse, Garnweidner-Holme et al. 2020 | Qualitative research using inductive analysis regarding acceptability of a tablet intervention  (Norway: Oslo) | Safe pregnancy study – Tablet intervention to prevent IPV, which included questions about IPV and a video.  Intervention materials have been translated into Norwegian, English, Urdu, and Somali. | Individual interview participants (N = 16). Experienced violence (6), no experience of violence (10)  Country of birth: Norway (9), Pakistan (3), Somalia (4)  Two focus group participants of professionals (community workers, social workers, and teachers).  Country of birth: Norway (3), Indian (1), Iran (1) recruited | Thematic analysis using semi-structured interview guide. | Women identified questions as sensitive, but relevant and easy to understand.  Safeguarding anonymity was important for women to disclose IPV.  Women reported that it was important to inform participant of all forms of abuse (physical and psychological).  Important was place on the effect of IPV on the child and the need to repeat information about safety behaviours. |
| Bacchus, Bullock, Sharps et al. 2016 | Qualitative interpretive study.  Purposive recruitment from RCT of DOVE intervention. (US: Virginia, urban and rural sites) | Domestic Violence Enhanced Home Visitation Program (DOVE) using mHealth technology  (ie, a computer tablet)  Vs Home visitor-administered, paper-based method | Women enrolled in the DOVE program  Paper-based (N= 18)  Computer tablet (N = 8)  White (46%), African American/black (31%), Other (23%).  Home visiting staff (N = 23)  DOVE designers (N = 2) | Narrative information collected via interview | Computer tablet viewed as safe and non-judgemental.  Some women and home visitors reported it helped connection whereas others felt it interrupted the relationship-based program.  Established trust and rapport and good interpersonal communication positively influenced comfort with either screening mechanism.  Technology helped reduce stigma of disclosing abuse.  Didactic video was limited, as it did not accommodate fluidity of women’s circumstances. |
| Klevens, Sadowski, Kee et al. 2012 | RCT  Recruitment from obstetrics, gynaecological and family planning clinics at a public hospital.  (US: Chicago) | Comparison of screening and referral strategies   1. HCP screen and   referral,   1. A-CASI screen and   referral with HCP referral endorsement   1. A-CASI screen and   referral plus video support  Health Care Providers (HCP)  Audio computer assisted self-interviews (A-CASI)] | Pregnant women.  HCP screen and referred (N = 46)  A-CASI screen and referred with HCP support (N = 37)  A-CASI screen and referral plus video support (N = 43)  White (6.3%), Black (78.6%), Latina (11.9%) Asian (3.2%) | Primary outcome was rate of IPV disclosure; secondary outcomes were screening mode preference, reactions to IPV screening, and use of referral  resources | A-CASI was more likely to result in higher rates of IPV disclosure and had similar rates of use of referral resources.  No women who reported IPV sought services within 3 months after screening. |
| Glass, Eden, Bloom et al. 2010 | Study recruitment from domestic violence shelters or support groups.  (US: Pacific Northwest) | Computerised Safety Decision Aid – one session. | Women living in shelters or connected to domestic violence support groups.  (N = 90)  White (61%), African American (17%), Native American/Alaskan (13%) | Gathered feedback after intervention completion with self-report survey | Safety decision aid:   - was useful and privacy was appreciated. - Helped women feel more supported and had less decisional conflict. |

**SBIRT and Multiple Health Risks and Protective Factors**

| **Study** | **Study Design** | **Intervention** | **Participants** | **Outcome Measures** | **Outcome** |
| --- | --- | --- | --- | --- | --- |
| Gance-Cleveland, Leiferman, Aldrich et al. 2019 | Qualitative analysis.  (US:Colorado) | SBIRT Startsmart - mobile health (mHealth) intervention to support evidence-based prenatal screening, brief intervention, and referral to treatment for risk and protective factors in pregnancy. | Pregnant women with history of anxiety, depression, substance use, gestational diabetes, overweight and/or IPV.  Clinicians included obstetricians, midwives and mental health providers.  Phase 1:  Two focus groups with 8 expert clinicians and scientists and six patient content experts.  Ongoing advisory work groups.  Phase 2:  Prenatal providers (N = 9) and patients (N = 7). | mHealth development approach where focus group and consultation data collected from patients  and providers informed the prototype development and refinement  of the technology to optimize the acceptability and  usability of the technology.  Prototype was then alpha tested by clinicians and patients. | Patients reported high acceptability and that tablet screening was useful to promote adherence to guidelines.  Clinicians reported mHealth facilitated capacity to provide comprehensive care. |

- RCT: Randomized Controlled Trial.
- SBIRT: Screening, Brief Intervention and Referral to Treatment.
- SR: Self-report.
- NICU: Neonatal Intensive Care Unit.
- C-BIAP: Computerized Brief Intervention for Alcohol Use in Pregnancy.
- ASSIST: World health Organisation (WHO) Alcohol, Smoking and Substance Involvement Screening.
- IPV: Intimate Partner Violence.
